# Hepatitis C prevalence in Denmark -an estimate based on multiple national registers

**DOI:** 10.1186/1471-2334-12-178

**Published:** 2012-08-06

**Authors:** Peer Brehm Christensen, Gordon Hay, Peter Jepsen, Lars Haukali Omland, Søren Andreas Just, Henrik Bygum Krarup, Nina Weis, Niels Obel, Susan Cowan

**Affiliations:** 1 Department of Infectious Diseases, Odense University Hospital, Building 1, 2 floor, Penthouse block 6, Sdr Boulevard, Odense C, 29 5000, Denmark; 2 School of Social and Political Sciences, University of Glasgow, Scotland, UK; 3 Department of Clinical Epidemiology, Aarhus University Hospital, Aarhus, Denmark; 4 Department of Infectious Diseases, Copenhagen University Hospital, Rigshospitalet, Denmark; 5 Department of Medicine, Svendborg Hospital, Svendborg, Denmark; 6 Department of Clinical Biochemistry, Aalborg University Hospital, Aalborg, Denmark; 7 Department of Infectious Diseases, Copenhagen University Hospital, Hvidovre, Denmark; 8 Department of Epidemiology, Statens Serum Institute, Copenhagen, Denmark

## Abstract

**Background:**

A national survey for chronic hepatitis C has not been performed in Denmark and the prevalence is unknown. Our aim was to estimate the prevalence of chronic hepatitis C from public registers and the proportion of these patients who received specialized healthcare.

**Methods:**

Patients with a diagnosis of chronic hepatitis C were identified from four national registers: a laboratory register, the Hospital Discharge Register, a clinical database of chronic viral hepatitis and the Register of Communicable Diseases. The total population diagnosed with hepatitis C was estimated by capture-recapture analysis. The population with undiagnosed hepatitis C was derived from the national register of drug users by comparing diagnosed and tested persons.

**Results:**

A total of 6,935 patients diagnosed with chronic hepatitis C were identified in the four registers and the estimated population diagnosed with the disease was 9,166 persons (95% C.I. interval 8,973 – 9,877), corresponding to 0.21% (95% CI 0.21%-0.23%) of the Danish population over 15years of age. The prevalence was highest among persons 40–49years old (0.39%) and males (0.28%). It was estimated that 40% of the diagnosed patients lived in the capital region, and 33.5% had attended specialised healthcare. It was estimated that 46% of hepatitis C patients had not been diagnosed and the total population with chronic hepatitis C in Denmark was 16,888 (95% C.I. 16,474-18,287), corresponding to 0.38% (95% CI 0.37-0.42) of the population over 15years of age.

**Conclusions:**

The estimated prevalence of chronic hepatitis C in Denmark was 0.38%. Less than half of the patients with chronic hepatitis C in Denmark have been identified and among these patients, one in three has attended specialised care.

## Background

Hepatitis C is not a common disease in Northern Europe, where most prevalence estimates in the general population are below one percent [1-3]. Hepatitis C is a blood borne viral infection, and in the Scandinavian countries the large majority of patients have been infected through drug injection. In Denmark 83% of reported transmissions in 2007 were due to injecting drug use and in Sweden 88% of 31,000 reported HCV transmissions between 1990 and 2006 were due to injecting drug use [4,5]. The reported prevalence of antibodies against hepatitis C virus (anti-HCV) among Danish injecting drug users was between 62% and 97% and half of them became infected within a year after the first injection [6-8].

Seroprevalence surveys of the general population are the gold standard for assessing the number of HCV infected within a country. This is time consuming and costly to perform and have never been done in Denmark. As the country has a long tradition of public registers and all residents have a unique civil registration number, the overlap between registers may be used to estimate the unregistered “hidden” population by capture-recapture analysis [9]. This type of analysis is often hampered by dependence between registers, but by using combinations of multiple sources interactions between registers may be identified and accounted for.

The primary aim of this study was to estimate the population with diagnosed and undiagnosed hepatitis C in Denmark by the use of national registers. Secondary aims were to estimate the proportion of patients who attended specialised clinical care and the coverage of the national registers for diagnosed cases of hepatitis C.

## Methods

### Settings

As of 1 January 2008, Denmark had a population of 5.5 million inhabitants [10]. Testing for hepatitis C was introduced in 1991 and medical care including treatment for hepatitis C has always been tax-paid and provided free-of-charge.

### Data sources

We used the unique 10-digit civil registration number assigned to all persons with permanent residence in Denmark to link the data sources described below. Persons without a valid civil registration number were excluded from the analysis.

The estimate was based on four national source registers:

Laboratory database (DANVIR): This research database included all patients tested for hepatitis C in 14 of the 18 Danish laboratories performing HCV tests (excluding blood donors). The laboratories contributing to the database served an estimated 85% of the Danish population and included data on 177,453 persons tested for hepatitis C. Data were included from the initiation of electronically stored test results used by all laboratories from 2000 onwards. From this register we included all subjects who had a positive HCV-RNA test not followed by a negative HCV-RNA. Thus patients who tested positive for anti-HCV, but had no HCV-RNA reported, were not included in the analysis.

Hospital Register: This was established in January 1977 (inpatients) and recorded all discharge diagnoses according to ICD-8 and ICD-10 codes, and since 1995 all hospital outpatient visits for patients treated in Danish hospitals. The International Classification of Diseases 8th revision was used until the end of 1993 and here after the 10th revision - ICD-9 was never used in Denmark.

From this register we extracted all individuals registered with chronic hepatitis C (ICD10 diagnosis B18.2).

Hepatitis database (DANHEP): This national clinical database contained all patients above 15 years of age with chronic viral hepatitis who attended specialist care in Denmark since 2002 regardless of the year of first contact. In 2009 it contained 6,489 persons. The database was updated once yearly and was complete as of 31.12.2007, the time of extraction. From this database we identified all patients with chronic hepatitis C (HCV-RNA positive at inclusion).

Communicable Diseases Register: This is a national public register of notifiable diseases and it contains mandatory report forms from the diagnosing physician of acute viral hepatitis C from 1991 and chronic hepatitis C from May 2000. The register is estimated to cover 35-40% of individuals diagnosed with chronic hepatitis C and contained a total of 8,202 persons with acute or chronic hepatitis B and C [11, 12]. From this register we included all patients reported with chronic hepatitis C.

Except for the laboratory register it was not possible to exclude patients who cleared the infection from the source registers. However spontaneous clearance of chronic HCV is less than 1%/year and in Denmark less than 2% of those infected have been cured by treatment [13, 14].

In addition to the four source registers we extracted data from:

Drug Treatment Register: This national register included all persons treated for drug abuse in Denmark since 1996 with detailed information of drug use, but no information of viral hepatitis. This register was used to estimate the population of drug users infected with hepatitis C that had not been diagnosed.

The Civil Register: This register was established in 1968 and stored information on vital status, residency as well as immigration and emigration on all Danish residents. From this register we extracted vital status and residency.

### Study population

In the study we included all individuals, who could be identified with chronic hepatitis C in one or more of the above four source registers. In order to assure equal chance of entrance into registers and to allow stratified geographical analysis we excluded all persons who were below 16years of age, had no assigned address or were reported dead, missing or emigrated in the civil register at 31.12.2007 Data were extracted from the above registers as of 31.12.2007.

We estimated the Danish population with chronic hepatitis C in a two step procedure.

**Figure 1:**
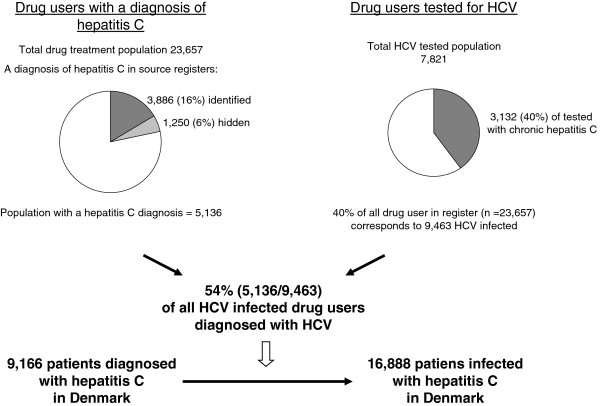
Calculation of the fraction of diagnosed cases and total Danish HCV estimate end 2007 from diagnosed and tested population in Danish drug treatment register.

1) The population with diagnosed chronic hepatitis C was calculated by capture-recapture analyses of the overlap between the four source registers stratified by gender, three age groups, five geographical regions and two time periods. This was based on log-linear modelling using the statistical program GLIM 4 [9, 15]. The final table contained 60 cells, and in total 113 different models including all possible two-way and three-way interactions were fitted to the overlap data. We primarily used the Akaike information criterion to select the ‘best’ fitting model, however when this model produced an estimate that differed markedly from the weighted estimate (averaged across all fitted model using the Schwartz criterion as a weight) then the Schwartz criteria was used to obtain the best fitting model. If there was discrepancy, or the choice between the Akaike and Schwartz criteria was not clear, then the model that produced an estimate closest to the ratio of known to estimated found in other strata was selected. Confidence intervals for the total estimate were derived from boot-strap analysis of 1000 samples [16, 17].

2) The proportion of patients with chronic hepatitis C that had not been diagnosed (never tested) was calculated from the drug treatment register. The number of diagnosed cases in the treatment register was extracted from the four source registers and increased by the estimated fraction of diagnosed cases not present in these registers (the “hidden” population) (Figure 1). We estimated the true proportion of HCV infected drug users in the drug treatment register as the proportion of HCVRNA positives divided by all drug users who had been tested for HCV. From this proportion we calculated the total number of HCV infected in the register, assuming the prevalence of hepatitis C was the same among tested and non-tested drug users. The diagnosed fraction in the drug treatment register was hereafter calculated as the estimated number of diagnosed patients divided by the estimated total number of hepatitis C patients in the register. Assuming the same proportion of diagnosed hepatitis C infection outside the treatment register we calculated the total prevalence of hepatitis C in Denmark from the capture-recapture estimate of diagnosed hepatitis C and the estimated coverage rate (Figure 1). A further boot-strap analysis, which also included a binomial distribution to account for the coverage rate, was used to obtain a 95% confidence interval of the total prevalence.Figure 1Calculation of the fraction of diagnosed cases and total Danish HCV estimate end 2007 from diagnosed and tested population in Danish drug treatment registerCalculation of the fraction of diagnosed cases and total Danish HCV estimate end 2007 from diagnosed and tested population in Danish drug treatment register

Statistical analysis was performed using SPSS v. 18.0. The study was approved by the Danish Data Protection Agency (J. 2008-41-2402).

## Results

### Estimate of population diagnosed with chronic hepatitis C

The initial extraction from the four source registers identified 9,315 individuals diagnosed with hepatitis C. We excluded 2,380 persons (25.5%) of whom 88% had died, 11% had unknown vital status (emigrants and missing persons), 1% were below 16years of age and 0.3% had unknown address. The remaining 6,935 cases were included in the capture recapture analysis (Table 1).

**Table 1:** Persons alive with chronic hepatitis C according to registers end 2007

	**Laboratory Database (DANVIR)**	**Communicable Diseases Register**	**Hospital Register**	**Hepatitis database (DANHEP)**	**TOTAL**
**Number**	3960	2890	4484	3065	6935
**% of total**	58.0	39.2	66.0	41.3	100
**Male (%)**	66.9	64.4	61.5	61.9	64.6
**Age in years (%)**					
<40	35.9	37.1	30.4	30.4	34.9
40-49	35.4	34.9	36.2	35.5	35.7
≥50	28.8	28.0	33.4	34.4	29.3
**Region (%)**					
North	10.8	5.0	9.0	11.1	9.0
Central	17.4	19.2	21.7	19.2	18.7
South	31.2	23.0	20.6	21.7	25.5
Zealand	10.2	13.6	11.4	8.8	11.5
Copenhagen	30.4	39.2	37.3	39.2	35.3
**Year of Inclusion (%)**					
≤2000	43.4	23.6	37.0	24.7	51.8*
>2000	56.6	76.4	63.0	75.3	48.2*
**Registered in drug treatment register (%)**	57.9	59.8	50.1	43.8	56.0

* first inclusion in any register.

Of the included cases 65% were men, but the proportion of men was lower in the clinical registers (hospital register and hepatitis database). These two registers also had a higher proportion of older people and a lower percentage of persons who had received treatment for drug use. In all registers most cases were included after the year 2000, and the geographical distribution was fairly constant between registers (Table 1).

The total estimate of persons alive with a diagnosis of hepatitis C was 9,166 cases (95% confidence interval 8,973 – 9,877) with a hidden population of 2,231 (24%) – these were individuals diagnosed with chronic hepatitis C but not identified in any of the source registers (Table 2). The estimate corresponded to 0.21% (95% C.I. 0.206%-0.226%) of the Danish population above 15years. The prevalence was highest amongst 40–49 year olds (0.39%), and was twice as high among men as among women (0.28% versus 0.14%). The regional prevalence ranged from 0.15% in the North region to 0.28% in Copenhagen, and the capital region represented 40% of all diagnosed cases. In all regions the age distribution changed over calendar time so that patients at inclusion were younger after 2000.

**Table 2:** Capture recapture estimates of patients diagnosed with chronic hepatitis C and alive end 2007

	**Region**	**North**		**Central**		**South**		**Zealand**		**Copenhagen**		**Total**		**Population**
		**n**	**%**	**n**	**%**	**n**	**%**	**n**	**%**	**n**	**%**	**n**	**%**	**prevalence**
**Age**	**<40**	250	36%	496	33%	1072	47%	357	34%	1171	32%	3346	37%	0.12%
	**40-49**	252	36%	537	36%	736	32%	339	32%	1331	37%	3195	35%	0.40%
	**50+**	194	28%	458	31%	496	22%	348	33%	1129	31%	2625	29%	0.13%
**Gender**	**Male**	463	67%	1019	68%	1524	66%	633	61%	2354	65%	5993	65%	0.22%
	**Female**	233	33%	472	32%	780	34%	411	39%	1277	35%	3173	35%	0.11%
**Year of entrance**	**≤2000**	407	58%	776	52%	1417	62%	454	43%	1324	36%	4378	48%	
**in 1. register**	**>2000**	289	42%	715	48%	887	38%	590	57%	2307	64%	4788	52%	
**Total**		696	100%	1491	100%	2304	100%	1044	100%	3631	100%	9166	100%	
**% of DK estimate**			8%		16%		25%		11%		40%		100%	
**Prevalence(%)**			0.12%		0.12%		0.19%		0.13%		0.22%		0.17%	
**Total pop. (in 1000)**		577		1,236		1,194		819		1,644		5,472		

The proportion of diagnosed cases present in each register varied from 31% in the communicable disease register to 49% in the hospital register, and 33.5% (3065/9166) had attended specialised clinical care (present in the hepatitis database DANHEP) (Table 1).

The stratified analyses covered 60 strata and the best models fitted to the stratified overlap patterns contained an average of two significant interactions between the four source registers. The most frequent interaction was between the hospital register and the communicable diseases register, and the hospital register and the clinical register; these were both found in more than half of the strata, whereas an interaction between the communicable disease and clinical registers was not found to be significant in any strata. By the inclusion of interaction terms the total estimate of patients with the hepatitis C diagnose increased from 7,012 to 9,166 patients.

### Estimate of the undiagnosed population with chronic hepatitis C

Among individuals alive and registered in the national drug treatment register, 33.1% (7,821/23,657) had a recorded test for anti-HCV and/or HCV-RNA in the laboratory register; 29.3% (2,293/7,821) had chronic hepatitis C (HCV-RNA positive), 15.3% (1,196) had past hepatitis C (anti-HCV positive and HCV-RNA negative), 38.1% (2,983) were unexposed (anti-HCV negative) and 17.3% (1,349) were anti-HCV positive, but had no recorded test result for HCV-RNA. In the laboratory register among all Danish patients with a positive anti-HCV and a test result for HCV-RNA, 62.2% (3,999/6,431; 95% CI 61.0-63.4%) were positive - indicating that 2/3 of Danish patients exposed to hepatitis C had developed chronic infection. Assuming that 62.2% (839/1,349) of anti-HCV positives in the drug treatment register not tested for HCV-RNA to be positive, the total prevalence of chronic hepatitis C among all tested drug users in the treatment register was 40.0% (3,132/7,821) (Figure 1). If 40% of the non-tested drug users in the register were also HCV-RNA positives, the total HCV-RNA positive population in the drug treatment register corresponded to 9,463 persons. The tested population was older (median age 40years/32years, p<0.001) and had a higher proportion of women (52% versus 40% p<0.001) than the not tested population. Drug users with hepatitis C were significantly older (43year/38years, p<0.001) whereas no gender difference was observed compared to drug users who did not have chronic hepatitis C.

In the drug treatment register 3,886 (16.4% of 23,657) had a diagnosis of hepatitis C in one or more of the four source registers (Table 1 and Figure 1). Adding the 24% unregistered cases (the hidden population derived from the national capture-recapture calculation) the total number of diagnosed cases increased to 5,136 (or 21.7% of 23,657) in the drug treatment register. Compared to the total of 9,463 patients with chronic hepatitis C estimated to be present in the drug treatment register, these 5,136 diagnosed cases represented 54.3% (95% CI 53.2%-55.3%).

We assumed the same diagnostic coverage (54.3%) among hepatitis C patients outside the drug treatment register. Including the 45.7% undiagnosed infections in the national estimate of hepatitis C, this rose from 9,166 to 16,888 (95% C.I. 16,474- 18,287), corresponding to a prevalence of chronic hepatitis C in Denmark of 0.38% (95% CI 0.37-0.42) of the population >15years of age.

Fifteen per cent of reported HCV transmissions in Denmark were not drug related, mostly nosocomial transmissions, and among these the diagnostic coverage may be different. Therefore we compared our estimates with a cohort of hepatitis C patients infected by blood transfusion and identified in the Danish hepatitis C look-back study [18]. Among 124 patients with diagnosed hepatitis C transmitted by transfusion 17% were not present in our source registers (compared to 24% in our estimate) and among a total of 187 recipients possibly infected (including 63 not tested) the four source registers identified 55% in agreement with the 54% estimate from the drug treatment register) (S Just, personal communication).

### Excluded populations

We excluded 749 patients from the laboratory register as they were initially HCV-RNA positive but later became negative. However, 564 of these were registered with chronic hepatitis C in one or more of the other registers – most likely representing resolved or cured infections, but reinfection was also possible and therefore they were kept in the analysis. Excluding these 564 individuals did not influence the estimate significantly (data not shown). We additionally excluded 4,928 persons who were positive for anti-HCV but never tested for HCV-RNA in the laboratory register. Including these persons from the laboratory register, as well as patients reported with acute hepatitis C (corresponding to all persons ever exposed to HCV) gave an estimate of 13,184 diagnosed cases. Assuming that only 62% of the added cases were chronic infections (HCV-RNA positives) the diagnosed population was 11,657, and corrected for undiagnosed patients, this corresponded to a total of 21,468 patients living with chronic hepatitis C in Denmark (0.49% of the adult population).

## Discussion

In this large register based capture-recapture analysis we estimated the population living with a diagnosis of chronic hepatitis C in Denmark to be 0.21% of the population. Including undiagnosed patients the total estimate of patients with chronic hepatitis C corresponded to 0.38% of the adult population. The higher prevalence among men, persons 40–49years of age and residents in the capital region was in agreement with previous population surveys and the geographic distribution of hepatitis C infected Danish blood donors [3,18,19]. The South region had the highest proportion of young men. Here systematic screening for hepatitis C had been performed amongst drug users since 1996. The clinical registers (hospital discharge register and the hepatitis database) had a lower prevalence of persons <40years of age, males and drug users in the treatment register. This could indicate that these groups had less clinical illness due to hepatitis C, or that young males were less likely to seek clinical care for hepatitis C. The latter explanation is in agreement with a recent Danish survey reporting that drug users diagnosed with clinically significant chronic hepatitis C were unlikely to enter hospital based clinical care [20]. It was surprising to us that only about a third of patients diagnosed with chronic hepatitis C attended specialised clinical care. With the improved therapies becoming available for hepatitis C these years, we suggest that new strategies must be developed in order to assure all patients with hepatitis C have proper care, once the disease has been diagnosed [21]. It was also unexpected that half of all patients were not present in the laboratory register as the hepatitis C diagnosis cannot be made without a positive HCVRNA test. There may be several explanations for this: HCV tests became available in 1991–2, but most laboratories did not import old test results when testing was computerised in the late nineties and we had only access to electronic test result. In addition, the participating laboratories only covered 85% of the Danish population.

Our study had several limitations: Capture-recapture analysis requires a closed study population and the same case definition in all registers. To fulfil this we excluded patients who died, and extracted data from all registers at the same day (31.12.2007), but we could not identify patients who cleared chronic hepatitis C, either spontaneously or by treatment except in the laboratory register. Spontaneous clearance of hepatitis C after development of chronic infection is rare and it is estimated that only 2% of hepatitis C patients in Denmark have been treated [13,14]. This bias could lead to overestimation of the hidden population. As for the case definition this may have changed over time. In the early nineties the clinical diagnosis had in some cases been based on positive anti-HCV (and elevated liver enzymes) in the communicable disease and hospital register. In contrast all cases in the laboratory and the hepatitis database (DANVIR and DANHEP) were based on positive HCV-RNA. Furthermore 9% of patients in the hepatitis database had cleared hepatitis C as a result of treatment [22]. With different case definitions between registers, the hidden population will be over estimated. On the other side, we excluded 4,928 anti-HCV positive persons from the laboratory register as these had no available HCV-RNA test, but it is likely that 62.2% (3064 patients 95%CI 3006–3124) of this group had chronic HCV infection. Our register estimate is lower than the general population estimates from Scandinavia, so most likely the bias mentioned let to an underestimation of the HCV infected population [2].

Another limitation was that the capture-recapture method only estimated the number of patients diagnosed with hepatitis C and not the population that had never been tested for HCV. A direct estimate would require a general population survey which had not been performed in Denmark. Instead, we estimated the test coverage within the population of hepatitis C infected in the drug treatment register and applied this (46% undiagnosed) to calculate the total population with chronic hepatitis C in Denmark. If the prevalence of hepatitis C was lower among the drug users who had not been tested, this could overestimate the hepatitis C population. As the drug users that had not been tested for hepatitis C were younger such a bias would be expected but an independent population survey, performed in 2007 among drug users in treatment in the county of Funen, found a 40% hepatitis C prevalence, very similar to what we estimated from the national drug treatment register [7]. Furthermore the hepatitis C prevalence among tested ever and never injectors in the treatment register were identical to the survey results (53% and 5%) giving further credibility to our estimate. An explanation of why an age difference did not correspond to a difference in hepatitis C prevalence could be that drug users becomes infected very rapidly after start of injection (50% within the first year) but on average do not enter treatment until after 4years of drug use and therefore the age difference is of less importance [7].

Another independent source was a national survey from 2004–2008, among 1009 drug related deaths, who were tested post mortem for hepatitis C. Their age and gender distribution was comparable to the drug treatment register (median age 38 years and 22% were female) and the estimated prevalence of chronic hepatitis C was 35% - slightly lower than our 40% treatment register estimate [23,24]. If the true hepatitis C prevalence was 35% in the treatment register, 62% would have been identified and the corresponding national estimate of hepatitis C infected would decline to 14,783 (0.34% of the adult population).

The estimates of test coverage for hepatitis C (54% and 62%) among drug users in Denmark used two different methods but both were higher than a 42% test coverage reported among drug users in Scotland, suggesting that our national hepatitis C prevalence estimates could be too low [25].

Another limitation in the above calculations was that we assumed the same test coverage among hepatitis C patients outside the drug treatment register (former and never injectors). We found practically the same diagnostic coverage for a cohort of never injectors (patients infected by blood transfusion), but a cohort of former drug injectors could not be identified.

A comparable hepatitis C estimate from England and Wales (using multiple surveys, register data and mathematical modelling) found that former injectors constituted 47%, current injectors 38% and never-injectors 15% of the total British hepatitis C estimate (0.60% of the adult population) [26,27]. Corresponding Danish estimates were 15-17% never users and 41% current injectors, deducing that former injectors may constitute 43% of the hepatitis C population [4,28,29]. A large proportion of former injectors with unknown but presumably low test coverage would make our estimate more uncertain and most likely too low. Our estimate was also lower than reported from two general population surveys from Sweden and Norway, both reporting an anti-HCV prevalence of 0.7% (corresponding to 0.45% and 0.5% with chronic hepatitis C infection) in the general population [2,30]. If the true Danish prevalence of hepatitis C infection was 0.45% (corresponding to 19,645 patients), and our estimate was too low due to lower test coverage among infected former drug users, these would constitute 51% of the estimate (10,050) and the diagnosed proportion with hepatitis C amongst former injectors would be 39% (3948).

## Conclusions

In conclusion we estimated the prevalence of chronic hepatitis C in the adult Danish population to be 0.38%, but due to bias and random variation the true prevalence could be within 0,34% - 0,49%. Of all patients with hepatitis C only half have been diagnosed and one third of the diagnosed attended specialised clinical care. This suggests that screening for hepatitis C should be improved and that new strategies are needed in order to deliver appropriate clinical care to the hepatitis C infected population. This will be of increasing importance as the treatment for hepatitis C continues to improve.

## Competing interests

The authors declare that they have no competing interests.

## Authors’ contribution

PBC contributed to the study design, collection and analysis of data and writing of the manuscript. GH performed the capture recapture analysis and revision of the manuscript. PJ, LHO, SAJ, HBK, NW, NO and SC provided source databases, participated in data analysis and revision of manuscript. All authors read and approved the final manuscript.

## Pre-publication history

The pre-publication history for this paper can be accessed here:

http://www.biomedcentral.com/1471-2334/12/178/prepub
